# *MRC2020*: improvements to *Ximdisp* and the MRC image-processing programs

**DOI:** 10.1107/S2052252523006309

**Published:** 2023-07-26

**Authors:** J. M. Short, C. M. Palmer, T. Burnley, M. D. Winn, Q. Zhang, B. V. Venkataram Prasad, S. Chen, R. A. Crowther, P. N. T. Unwin, R. Henderson

**Affiliations:** a MRC Laboratory of Molecular Biology, Cambridge CB2 0QH, United Kingdom; b Science & Technology Facilities Council, Research Complex at Harwell, Harwell, Didcot OX11 0FA, United Kingdom; c Sun Yat Sen University, School of Life Science, State Key Laboratory of Biocontrol, Guangzhou 510275, People’s Republic of China; d Verna and Marrs McLean Department of Biochemistry and Molecular Pharmacology, Baylor College of Medicine, Houston, TX 77030, USA; Boston University School of Medicine, USA

**Keywords:** *MRC2020*, image-processing programs, cryoEM

## Abstract

The great success of single-particle electron cryo-microscopy (cryoEM) during the last decade has involved the development of powerful new computer programs and packages that guide the user along a recommended processing workflow, in which the wisdom and choices made by the developers help everyone, especially new users, to obtain excellent results. The ability to carry out novel, non-standard or unusual combinations of image-processing steps is sometimes compromised by the convenience of a standard procedure. Some of the older programs were written with great flexibility and are still very valuable. Among these, the original MRC image-processing programs for structure determination by 2D crystal and helical processing alongside general-purpose utility programs such as *Ximdisp*, *label*, *imedit* and *twofile* are still available. This work describes an updated version of the MRC software package (*MRC2020*) that is freely available from CCP-EM. It includes new features and improvements such as extensions to the MRC format that retain the versatility of the package and make it particularly useful for testing novel computational procedures in cryoEM.

## Abstract 2

An update to the MRC image-processing software package is described. It includes improvements to the *Ximdisp* display program for use on images of helical assemblies or tilt-pair single-particle cryoEM images.

## 1. Introduction

There has been a revolution in the structural determination of biological complexes by electron cryo-microscopy (cryoEM) over the last fifty years. Advances in specimen grid technology, microscopes, cryo-stages, detectors, processing software and automation of all these procedures have combined to allow superb images to be obtained showing structures at resolutions that rival both X-ray and electron crystallography. The introduction of maximum likelihood methods into the software used for single-particle analysis has been crucial to this success and underpins the most recent advances. The earlier program packages such as *Spider* and *Imagic*, as well as the original MRC image-processing programs for structure determination by 2D crystal and helical processing together with utility programs such as *Ximdisp*, *label*, *imedit* and *twofile* are still available. Though these programs offer great flexibility for many purposes, this is accompanied by less convenience for pursuing the streamlined path that many users prefer. The MRC program package has been widely distributed since its inception with contributions from many authors. During that time, changes in computers, compilers and operating systems together with recent extensions to data formats have necessitated program updates, including extensions to the MRC format agreed in 2014. These improvements and extensions are described in this paper.

## 2. Historical background

The computer programs for the first reconstruction of 3D helical structures (DeRosier & Klug, 1968 ▸) were described earlier (DeRosier & Moore, 1970 ▸). At that time, specimens for microscopy were negatively stained and the resulting micrographs recorded on film which was then densitometered to produce a digitized image. Central to the computational analysis of the images were programs for the calculation of Fourier transforms, based on the Cooley–Tukey algorithm (Cooley & Tukey, 1965 ▸). These programs provided the foundation for the MRC image-processing package (Crowther *et al.*, 1996 ▸). The original suite of programs, written in Fortran, were designed specifically for the analysis and calculation of helical structures and resulted in a 3D density map. Following the helical programs, further software was written, also in Fortran, for analysis of images of icosahedral viruses (Crowther *et al.*, 1970 ▸, 1971 ▸), and subsequently yet more for analysis of electron diffraction data and micrographs of 2D crystal specimens (Henderson *et al.*, 1986 ▸).

It became clear that a general-purpose format for the storage and computation of digitized images and Fourier transforms was necessary. The format, known as MAPFORMAT, was originally designed in 1982 by David Agard and Phil Evans for handling crystallographic density maps. The MRC format was derived from MAPFORMAT with the specific purpose of storing electron microscope density maps as well as Fourier transforms. The format incorporated a 1024 byte header, carrying information about the file, such as the number of pixels in *x*, *y*, *z* mode for data type *etc*. This was (optionally) followed by 80 bytes or more of symmetry data before writing the image density as single-byte, signed 2 byte integers or 4 byte floating point data. Fourier transforms were written as complex*4 or complex*8 and the phase origin was stored in the header. Utility programs *label*, *imedit*, *twofile*, *padbox*, *Ximdisp* and others were added over the years.

As in crystallography, the original programs in the MRC image-processing suite, *helical*, *icosahedral* or *2D crystal*, were limited to structure determination that made use of the symmetry of the specimen. However, the technique of single-particle analysis, which entirely avoids this restriction, originally embodied in packages such as *SPIDER* (Frank *et al.*, 1981 ▸; Shaikh *et al.*, 2008 ▸) and *IMAGIC* (van Heel & Keegstra, 1981 ▸; van Heel *et al.*, 1996 ▸), was also under development. More recently, other packages such as *RELION* (Scheres, 2012 ▸), *XMIPP* (Sorzano *et al.*, 2004 ▸), *FREALIGN* (Grigorieff, 2016 ▸), *CryoSPARC* (Punjani *et al.*, 2017 ▸), *EMAN* (Ludtke *et al.*, 1999 ▸), *SPHIRE* or *SPARX* for high-resolution electron microscopy (Wagner *et al.*, 2019 ▸), *cisTEM* (Grant *et al.*, 2018 ▸), and others have revolutionized structure determination. At the same time, innovations of specimen preparation by plunge-freezing (Dubochet *et al.*, 1988 ▸) and the revolution in microscopes (Nakane *et al.*, 2020 ▸), grid (Russo & Passmore, 2016 ▸) and detector development (McMullan *et al.*, 2014 ▸) have brought cryoEM in line with crystallography in terms of resolution. Furthermore, it has provided the ability to determine structures of large, flexible biological complexes and many membrane proteins or membrane protein complexes that have proved very difficult to crystallize.

However, since microscopes now collect a series of frames (movies) for each recorded micrograph image, the demand for computing power and data storage has increased dramatically. To meet these demands, microscope manufacturers and users have economized data storage by adopting 2 byte unsigned integers as the preferred recording format, which is mode 6 in the MRC format. Added to this modification, they also store new information (metadata) between the 1024 byte header and the multi-frame image intensity data. These extensions of the original format are described in a paper on *MRC2014* (Cheng *et al.*, 2015 ▸). In addition, there is now mode 12 [16-bit float (IEEE754)], a new feature introduced in *RELION* (version 4.0) to save a factor of two in required disc space (Kimanius *et al.*, 2021 ▸); and mode 101 (4-bit data packed two per byte), proposed by Agard and Mastronarde in 2015 for use in *SerialEM* (Schorb *et al.*, 2019 ▸). Neither of these extensions are included in *MRC2020*. Since the first release of the MRC package over 50 years ago, computers, operating system requirements and compilers have also changed. To accommodate all these changes, along with new user requests, we therefore updated and extended all the MRC programs and present here the 2020 version of the MRC image-processing package.

## 3. Overview

Our earlier paper (Crowther *et al.*, 1996 ▸) described in detail the functionality of each of the applications programs included in the package. Programs accessing the MRC data files are linked with libraries providing the I/O: *imsubs* (now *imsubs2020*), written in Fortran, provides the interface to a set of CCP4 routines, written in C, which themselves directly read/write the MRC files (Winn *et al.*, 2002 ▸). Some programs also require libraries such as *ifftlib*, which provides the Fourier transform calculations, and *plot2klib* for plotting output. All of these are included in the package. The graphics display program *Ximdisp* (Smith, 1999 ▸) requires X11/R5 or higher and the *Athena* widget set *Xaw*, and calls the Fortran interface library *Ximagelibf.for*, which accesses *Ximagelibc.c*, written in C. Other libraries, included in the distributed package, are also required by this program.

## 4. Changes to computers, operating systems and compilers

Before the year 2000, endianness varied between different computer systems. Big-endian CPUs are now rare but at the time it was necessary for the programs, when reading a file, to determine the endianness of the computer which wrote the file. The ability to ascertain endianness is a feature of the subroutine package *imsubs2020*, which continues to determine the architecture of the machine that wrote the file and proceeds accordingly, printing an error message if the architecture is incompatible with the CPU running the program. Around the same time that this feature was installed, it emerged that CCP4 had decided to include a machine stamp in the header to allow software reading the file to determine endianness. However, the machine stamp was written in the same position as the MRC programs used to store the phase origin for Fourier transforms. The phase origin was then moved to a new position where it currently remains.

## 5. Program modifications

**Figure 1:**
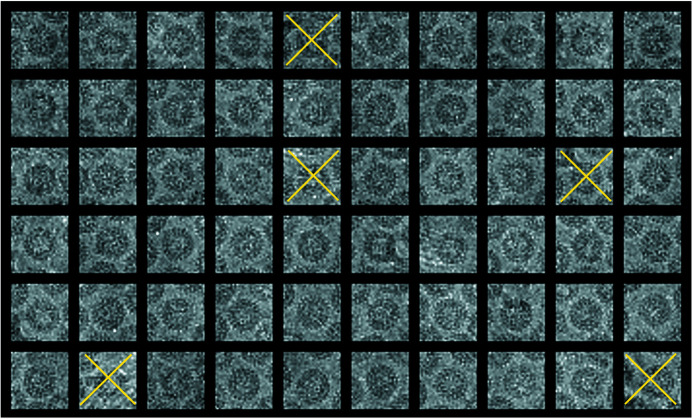
Multi-image editing from a stack of images in *Ximdisp*: a gallery of images can be displayed from a multi-stack file. Poor quality images can be removed manually and are marked by a cross (yellow). On completion an output stack file is written, with the marked images removed and a list of their numbers in a separate file. The illustration shows hepatitis B virus core particles (see Böttcher *et al.*, 1997 ▸; and EMD-13734).

**Figure 2:**
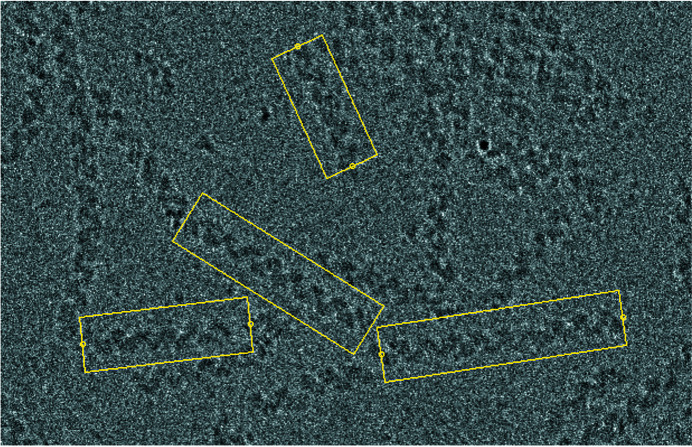
Filament boxing option of *Ximdisp*. The user sets the box width before selecting each end of each candidate filament. Box corners are stored in a file which can be read by a separate program for calculating box centres with a user set inter-box distance. The box centres are stored in a file which can be read directly into *RELION* for processing. The illustration shows HsRad51 complexed with single-stranded DNA (EMD-8183; Short *et al.*, 2016 ▸).

**Figure 3:**
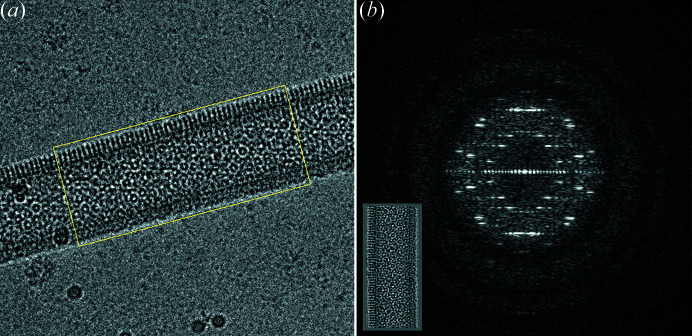
Interactive display of Fourier transforms from helical structures in *Ximdisp*. In this mode, the filament is boxed (*a*), then vertically aligned (inset) before calculation and display (*b*) of the transform. It is possible to sample the quality of various parts of the filament prior to selection by moving the box and re-calculating the transform. The illustration shows an acetyl­choline receptor tube (see Unwin, 2005 ▸; and EMD-11239).

Many changes to the programs represent bug fixes. Most were flagged by newer, more stringent compilers and have been corrected. The helical programs have been extended to provide the ability to work on bigger Fourier transforms up to 4096 × 4096 (Short *et al.*, 2013 ▸). These programs continue to be useful for calculating starting models and helical parameters from structures to be calculated by single-particle processing. The 2D crystal processing programs have also been maintained in their original form (Henderson *et al.*, 1990 ▸), but many of them have been incorporated into or improved in the *2dx* package (Gipson *et al.*, 2007 ▸). *Ximdisp* (Smith, 1999 ▸) has been extensively modified and now includes editing a stack of images (Böttcher *et al.*, 1997 ▸) displayed as a gallery (Fig. 1 ▸), boxing of helical filaments (Short *et al.*, 2016 ▸) (Fig. 2 ▸), improvements to spline fitting a helical filament, new colour tables for display, interactive Fourier transform display from any section from a stack using the scrolling section option and numerous other options. The interactive Fourier transform option is useful for assessing the quality of the image and for selecting the best diffracting stretches of a helical filament, (for example, Fig. 3 ▸, which shows the transform of part of a filament of the acetylcholine receptor (Unwin, 2005 ▸). Another extension to *Ximdisp*, which facilitates particle picking for tilt-pair analysis, is described in the next section.

## 6. Tilt-pair particle picking

**Figure 4:**
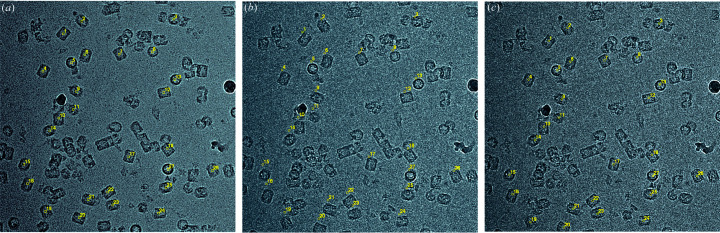
Picking particles from tilted pairs in *Ximdisp*. The specimen imaged in this example is *Haliotis diversicolor* hemocyanin, molecular weight 8 MDa (EMD-5585, EMD-5586; Zhang *et al.*, 2013 ▸). Images were recorded on film at 200 keV. *Ximdisp* was used to pick particles manually from the left image (*a*) which was recorded with a specimen tilt angle of −5°. This was then transferred by *Ximdisp* to the middle image (*b*) which was recorded at a tilt angle of +5°. The positions of a few particles in the middle image were corrected manually and the shifts calculated by *Ximdisp* were used to correct the remaining particles automatically, as seen in the right image (*c*). A plot was obtained after alignment of both −5 and +5° sets of particles to the starting model and the tilt axis and angle describing the geometrical relationship between the two sets of particles calculated. The resulting tilt-pair plot is shown in Fig. 5 ▸(*a*).

**Figure 5:**
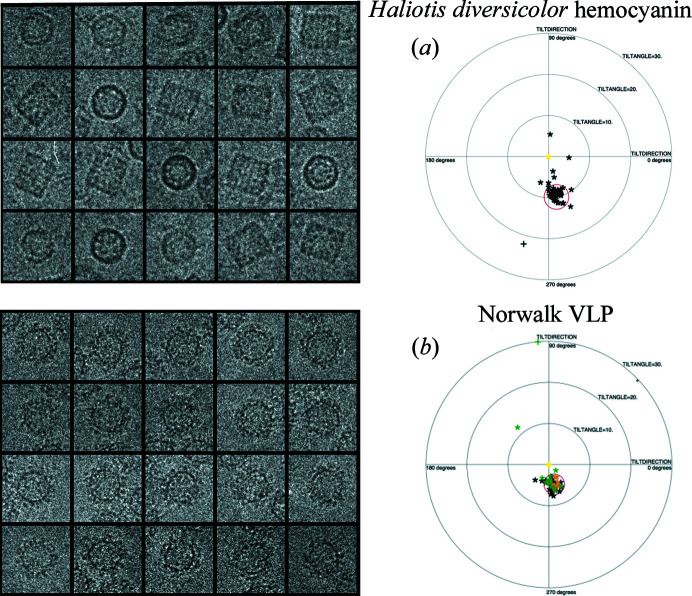
Examples of tilt-pair plots from two different samples, used to validate determination of the single-particle orientations that are needed to calculate the 3D structure. In (*a*) the change in the angle of the *Haliotis diversicolor* hemocyanin particles is clustered at 10°, which was the angular difference between the two images, indicating the validity of the 3D model. The structure of this hemocyanin was solved subsequently to a resolution of 4.5 Å (Zhang *et al.*, 2013 ▸). In (*b*) a similar experiment on Norwalk virus-like particles with a 5° change in tilt angle between the two images is shown. In this experiment, three pairs of images were recorded, with particles from different image pairs shown in different colours. With molecular weights of 8 and 10 MDa, respectively, these two additional tilt-pair parameter plots fill in the gap in particle size, between 3 and 50 MDa, that was missing from an earlier publication (PDB code 1ihm; Prasad *et al.*, 1999 ▸) on a range of different specimens.

Analysis of two images recorded from the same particle or particles at two different specimen tilt angles was proposed (Rosenthal & Henderson, 2003 ▸) as a way to validate and optimize determination of the angular parameters in single-particle cryoEM, and to establish absolute handedness. Its use was demonstrated on a single specimen, the catalytic domain of icosahedral pyruvate de­hydrogenase. Its value was subsequently extended to a range of different specimens of varied size and symmetry (Henderson *et al.*, 2011 ▸), which showed that the accuracy of particle orientations increased with the particle size, but there was a size gap between 3.5 and 50 MDa. To fill this gap, we have used the new particle-picking feature in *Ximdisp* to carry out tilt-pair analysis of two new samples with molecular weights of 8 and 10 MDa. The first is *Haliotis diversicolor* hemocyanin (Zhang *et al.*, 2013 ▸), with molecular weight 8 MDa and *D*_5_ symmetry (Fig. 4 ▸, Fig. 5 ▸). The second is Norwalk virus-like particles (Prasad *et al.*, 1999 ▸), with molecular weight 10 MDa and icosahedral symmetry (Fig. 5 ▸). Together these two new tilt-pair plots (Fig. 5 ▸) confirm the trend (Henderson *et al.*, 2011 ▸) that the accuracy of angular orientation determination improves with the size of the particle. The tilt-pair particle-picking feature in *Ximdisp* provides a complementary increase in ease of use to that provided by the tilt-pair web server (Wasilewski & Rosenthal, 2014 ▸).

## 7. New additions

Several new utility programs have been added, including *interpo3D* which reinterpolates a 3D map involving rotation, translation or magnification change, and *swapxy* which changes the handedness of 2D or 3D images and maps. Others such as *taperedge* which smoothly equalizes the densities along the boundaries, so that Fourier transforms do not show artefactual stripes along the axes, have been improved. An updated list of the MRC programs is available in Table S1 of the supporting information.

## 8. Conclusions

Additions, extensions and enhancements to what could otherwise be described as a mature, legacy package of MRC image-processing programs have proved to be useful in applications where novel combinations of steps are needed. Each program can be used on its own for simple stand-alone tasks, or as part of a scripted combination without integration into a larger suite, thus providing a flexible toolkit for new developments. The *MRC2020* software package is freely available under an open-source licence (BSD) from the CCP-EM website at https://www.ccpem.ac.uk. Typically, there have been around 50 downloads of the *MRC2020* package per year during the last 2 years. CCP-EM also maintains the ‘mrcfile’ Python library for reading, writing and validating MRC data files. A description of the full software framework for cryoEM managed by CCP-EM, including the MRC image-processing package that is the topic of this publication, is given by Burnley *et al.* (2017 ▸).

## Supplementary Material

Table S1. DOI: 10.1107/S2052252523006309/eh5017sup1.pdf
