# Supplementary material for: MRC2020: improvements to Ximdisp and the MRC image-processing programs
Source: IUCrJ. 2023 Jul 26;10(Pt 5):579–83. doi: 10.1107/S2052252523006309 (PMC10478516; doi:10.1107/S2052252523006309)
Supplement: Supplementary file 1 [file m-10-00579-sup1.pdf]

IUCrJ

Volume 10 (2023)

Supporting information for article:

MRC2020: improvements to *Ximdisp* and the MRC image-processing programs

J. M. Short, C. M. Palmer, T. Burnley, M. D. Winn, Q. Zhang, B. V. Venkataram Prasad, S. Chen, R. A. Crowther, P. N. T. Unwin and R. Henderson

TABLE I : Updated brief description of MRC programs

Densitometry

1. NIKSCN **	Digitization using NIKON densitometer.
2. JLSCAN **	Digitization using JOYCE-LOEBL densitometer.

General processing

1. HEADER	Print out information in header records.
2. IMCON **	Convert raw densitometer output into MAPFORMAT.
3. LABEL	Image handling of various kinds.
4. FFTRANS	Fast Fourier transform.
5. TRNOUT	Output amplitudes and phases to printer.
6. TRMASK	Mask transform for filtering.
7. INTERPO	General 2D reinterpolation program.
8. INTERPO3D	3D version of INTERPO.
9. SWAPXY	Swap XY axes of a 3D or 2D image or stack or images.
10. BOXIM	Standard boxing program for selection of an area.
11. BOXIMAGE	Similar to above, but leave boxed area in original position.
12. IMEDIT	General editing program for image headers.
13. MEDIAN **	Median filtering for smoothing images.
14. ENHANCE **	Apply various radial filter functions to transforms.
15. IMEXCHANGE **	Convert MAPFORMAT into ASCII for editing/exchange.
16. REMORIG	Remove origin with taper for crude high-pass filtering.
17. TAPEREDGEK	Taper edge of an image to remove spikes in transform.
18. TWOFILE	Linear combination, or multiply/divide data in two files.
19. TIF2MRC	Convert TIFF format files to MAPFORMAT.
20. MRC2TIF	Convert MRC format files to TIFF.
21. SPIDERTOMRC **	Convert SPIDER format files to MAPFORMAT.
22. JOINSTK_V1_00	Joins multiple image stacks.
23. BANDPASS	High/low bandpass image filtration with selection of smooth function cutoffs.
24. IMAGE_CONVERT	converts old style (pre-2000) maps to new post 2000 format
25. BYTE_SWAP_MAP	Byte swaps map data to change map enddianness.
26. CTFCALCK	Calculate and plot CTF curves.
27. CTFFIND2	Predecessor of Grigorieff CTFFIND3 and CTFFIND4
28. CTFFINDA	Predecessor of CTFFIND2

29. PADBOX	Pads an image or map with zeroes.
30. PADCORCUT	Pads an image, applies CTF then cuts to original box size.
31. SMOOTH	Smoothing by 3x3 local averaging.

Two-dimensional crystals

1. EMTILT	Calculate tilt angles from lattice parameters.
2. MASKTRANA	Mask transform for filtering, like TRMASK.
3. AUTOCORRL	Autocorrelation calc + expansion 2 use with QUADSERCH.
4. QUADSERCHK	Correlation peak search on lattice, with profile fit.
5. CCUNBENDK	Unbend image using list of peaks from QUADSERCH.
6. MMBOXA	Read amplitudes and phases from transform and display surrounding box.
7. MMLATREF	Lattice parameter refinement based on MMBOX.
8. TTBOXK	Correct for tilted transfer function, gives amplitudes and phases.
9. TTMASK	Combined MASKTRAN and TTBOX, masking + TTF corr on tilted images.
10. TTREFINE	Refine defocus, astigmatism, tilt params on tilted images.
11. TTBOXREF **	Lattice parameter refinement on images with TTF correction.
12. CTFSEARCH	Refine or search for correct defocus, astigmatism, on untilted images.
13. CTFAPPLYK	Apply CTF to data from MMBOX, with graphical output.
14. ORIGTILTK	Combine data from different images using crystal symmetry.
15. LLFILT **	Lattice line fitting for smooth curves and structure factors.
16. LATLINEK	Agard's least squares latline fit of amplitudes and phases.
17. ALLSPACEA	Determine space group, origin, beamtilt for single image.
18. AVRGPHASES **	Overall averaging of projection data from ORIGTILT.
19. AVRGAMPHS	Overall averaging of amplitude and phase projection data.
20. MAKETRAN	Create reference transform from MTZ file with given defocus.
21. SCALIMAMP3D	Scales image amplitudes to selected reference data.
22. LATLINPRESCAL	Corrects image amplitudes for CTF.
23 MMTOMKLCF	Creates MKLCF file from MMBOX or CTFAPPLY.
24. PICKPROFK	Spot integration, using least-squares profile-fitting.
25. PLTILTK	Plot tilt geometry distribution.
26. PLTSYNCP3	Plot output from SYNCFITP3.
27. PREPMKLCF	Prepare latline output for MKLCF.
28. TWOLATTK	Plots two lattices to see if there is overlap.
29. DIVIDEQ	Divides merged list of amps and phases into two equal halves.
30. HALFSTAT	Calculates phase difference between two halves of data.

Electron diffraction patterns

1. BACKAUTOK	Calculate radial background and find centre of pattern.
2. AUTOINDEXK	Find two simplest lattice vectors automatically.
3. PICKAUTOK	Integrate and background correct electron diffraction spots.
4. MERGEDIFF	Merge e.d. data and do a host of corrections.
5. AVRGFDELF	Average multiple measurements of delta-F from MERGEDIFF.
6. SYNCFITP3	Fit lattice line curves to output from MERGEDIFF.
7. F2MTZ **	Convert formatted data (A,P) to MTZ format (CCP4 Suite).
8. AVRGINTENS	Overall averaging of electron diffraction intensities in projection.

Helical structures

1. HLXSEARCH	Determine tilt and origin.
2. HLXDUMP	Dump layer line data from transform.
3. HLXFIT	Orientations and origins of different particles.
4. HLXFITT	Updated version of HLXFIT.
5. HLXAVG	Average data from different particles.
6. HLXFOUR	Fourier program
7. HLXPROJ	2D filtered image from layer line data.
8. EM2FOLD	Impose twofold normal to axis.
9. HLXSEPDAT	Feeder for HLXSEPR.
10. HLXSEPR	Separate overlapping Bessel functions.
11. HLXLLOUT	Graph standard layer line data.
12. HLXSIMUL	Simulate image of helix.
13. HCUT	Removes layer lines outside defined resolution range.
14. LLGRAPH	Plots layer line data.

Icosahedral viruses

1. REFINE **	Find orientations and origins by self-common lines.
2. X60CORR **	Cross common lines between two particles of known orientation to find relative hand and scaling.
3. RACMAT **	Extract data from transforms needed for solving for big G's.
4. RACBG **	Solve for big G's.
5. RACLG **	Convert big G's to little g's.
6. RACFB **	Fourier summation from little g's to make 3D map.
7. MAPCONV **	Convert 3D Fourier map to MRC format.
8. PROJGEN **	Compute general projection of 3D map.
9. X60REF **	Find orientations and origins by cross-common lines between unknown particle and projections of current best model.

10. SHELLS **	Section map on spherical shells for display

Rotational averaging and filtering

1. RFILTIM	Rotational filtering.
2. ROTAV	Rotational averaging of 2D images or 3D maps about z axis.
3. IMROTRAN	Rotate/translate 2D image—search to maximize correlation.

Calculating and contouring maps (from CCP4 suite)

1. FFT	Crystallographic Fourier program.
2. EXTEND	Extend maps/images to multiple unit cells.
3. PLUTO	General contouring, atomic model plotting program.

General display

1. LASERTONE	General program for conversion of image to postscript file.
2. LASERPLOT	General program for conversion of plot file to postscript file.
3. LASERTEXT	General program for conversion of text to postscript file.
4. HISTOK	Make histogram of densities in an image.
5. CURVY2K	Product graph on line printer.
6. Ximdisp	LMB raster graphics display for X-terminals.
7. THREED	Display 2D array as simulated 3D contoured surface.
8. SURF, LIGHT	Produce shaded surface representations of 3-D maps.

** These programs are now obsolete or not included in the current distribution via CCP-EM. They may still be available from the authors of Crowther et al (1996).
